# The Rating of Voluntary Hospitals

**Published:** 1897-09-11

**Authors:** 


### THE RATING OF VOLUNTARY HOSPITALS.

The question as to whether hospitals should pay local rates is one which has engaged the attention of Parliament and hospital managers for thirty years past. Whatever may be the opinion held on the general question, no one will be found to deny that if hospitals are to be charged local rates, such charges should be made on some uniform and definite principle. Mr. Thomas Ryan, the able secretary of St. Mary's Hospital, has taken a great deal of trouble to ascertain the actual rates paid by several important provincial hospitals, and the facts he has elicited are clearly set forth in the following table. It will be observed that the figures relate to the year 1895.

It is manifestly indefensible that a great hospital like the Leeds General Infirmary, where the number of occupied beds amounts on an average to 360, should be rated at 6s. 8¾d. in the pound, and the Leicester Infirmary with 196 occupied beds, the Bristol Royal Infirmary with 199 occupied beds, and the Bristol General Hospital with 172 occupied beds, should be rated at 6s. 4d. in the pound; whilst the Birmingham General Hospital with 230 occupied beds, the Adden-

Return of the Local Rates Paid by Fifty-four of the Principal Provincial Hospitals in England. (1895.)

| Name of Hospital. | No. of Beds. | Average No. occupied daily. | Rateable Value. Gross. £ s. d. | Rateable Value. Nett. £ s. d. | Rate per £ per annum in 1895. s. d. | Amount Paid in 1895. £ s. d. |
|---|---|---|---|---|---|---|
| **Hospitals with Medical Schools—** | | | | | | |
| Leeds General Infirmary ... ... ... ... ... ... | 394 | 360 | 633 5 0 | 633 5 0 | 6 8¾ | 214 0 0 |
| Birmingham General Hospital ... ... ... ... | 271 | 230 | 307 0 0 | 291 0 0 | 4 6 | 65 9 6 |
| Newcastle-on-Tyne Royal Infirmary ... ... ... | 272 | 267 | 100 0 0 | 88 0 0 | 5 0½ | 22 3 8 |
| Bristol Royal Infirmary ... ... ... ... ... | 270 | 199 | — | 311 0 0 | 6 4½ | 99 2 7 |
| Sheffield General Infirmary ... ... ... ... | 200 | 185 | 376 0 0 | 282 10 0 | 8 2 | 114 18 2 |
| Liverpool Royal Southern Hospital ... ... ... | — | — | Not | Rated. | — | — |
| Bristol General Hospital ... ... ... ... ... | 200 | 172 | — | 245 0 0 | 6 4¾ | 78 11 5 |
| Addenbrooke's Hospital, Cambridge ... ... ... | 153 | 106 | 320 0 0 | 240 0 0 | 4 0 | 48 0 0 |
| Radcliffe Infirmary, Oxford ... ... ... ... | 139 | 106 | 580 0 0 | 464 0 0 | 3 7 | 83 2 8 |
| Queen's Hospital, Birmingham ... ... ... ... | 132 | 115 | 168 0 0 | 143 0 0 | 4 6 | 32 3 6 |
| **Hospitals without Medical Schools—** | | | | | | |
| Leicester Infirmary... ... ... ... ... ... | 246 | 196½ | 400 0 0 | 300 0 0 | 6 4 | 95 0 0 |
| Wolverhampton Hospital ... ... ... ... ... | 230 | 138 | 562 0 0 | 450 0 0 | 7 3 | 163 2 6 |
| North Staffs Infirmary ... ... ... ... ... | 222 | 179 | Not | Rated. | — | — |
| Norfolk and Norwich Hospital ... ... ... ... | 220 | 134 | *500 0 0 | 450 0 0 | 8 8 | 195 0 0 |
| Bradford Infirmary... ... ... ... ... ... | 220 | 173 | — | 323 0 0 | 4 6 | 72 13 6 |
| Devon and Exeter Hospital ... ... ... ... | 214 | 160 | — | 289 0 0 | 5 7 | 80 13 7 |
| Sunderland Infirmary ... ... ... ... ... | 210 | 180 | Not | Rated. | — | — |
| Sussex County Hospital ... ... ... ... ... | 110 | 152 | — | 50 0 0 | 5 2½ | 13 0 5 |
| Hull Royal Infirmary ... ... ... ... ... | 180 | 142 | — | 40 0 0 | 7 1 | 14 3 4 |
| Notts General Hospital ... ... ... ... ... | 175 | 157 | — | 42 0 0 | 6 5 | 13 9 6 |
| Bath Royal Mineral Water Hospital ... ... ... | 171 | 150 | 450 0 0 | 382 10 0 | 4 3 | 81 5 7 |
| Gloucester General Infirmary ... ... ... ... | 156 | 92 | 172 0 0 | 146 0 0 | 4 4 | 31 7 10 |
| Chester General Infirmary ... ... ... ... | 163 | 104 | Not | Rated. | — | — |
| Salop Infirmary ... ... ... ... ... ... | 115 | 90 | 339 0 0 | 296 15 0 | 2 6 | 37 1 10 |
| York County Hospital ... ... ... ... ... | 140 | 104 | — | 16 0 0 | 6 7¾ | 5 6 4 |
| Swansea General Hospital... ... ... ... ... | 130 | 88 | — | 100 0 0 | 9 5¼ | 47 4 2 |
| Worcester General Infirmary ... ... ... ... | 127 | 88 | — | 170 0 0 | 5 6 | 46 15 0 |
| Lincoln County Hospital ... ... ... ... ... | 125 | 86 | — | 42 3 0 | 5 2 | 10 17 10 |
| Derbyshire Royal Infirmary ... ... ... ... | 159 | 120 | — | 1,021 0 0 | 2 6 | 127 12 6 |
| East Suffolk and Ipswich Hospital ... ... ... | 110 | 83 | 230 0 0 | 207 0 0 | 6 0 | 62 2 0 |
| Salisbury Infirmary ... ... ... ... ... | 110 | 96 | 92 0 0 | 79 0 0 | 4 4½ | 17 4 6 |
| Bolton Infirmary ... ... ... ... ... ... | 107 | 87 | — | 40 0 0 | 5 3¾ | 10 12 6 |
| Hereford General Infirmary ... ... ... ... | 107 | 60 | — | 51 7 6 | 4 3 | 10 18 4 |
| Kent and Canterbury Hospital ... ... ... ... | 106 | 69 | 200 0 0 | 160 0 0 | 5 0 | 40 0 0 |
| Bootle Borough Hospital ... ... ... ... ... | 103 | 65 | — | 80 0 0 | 2 11 | 11 13 4 |
| Blackburn Infirmary ... ... ... ... ... | 102 | — | Not | Rated. | — | — |
| Taunton and Somerset Hospital ... ... ... ... | 100 | 78 | — | — | 4 1 | 28 19 1 |
| Royal Halifax Infirmary ... ... ... ... ... | | | Exempt by Act of Parliament. | | | — |
| Bedford General Infirmary and Fever Hospital ... | 110 | 60 | — | 184 0 0 | 5 4 | 49 1 4 |
| Royal Albert Hospital, Devonport ... ... ... | 92 | 72 | Not | Rated. | — | — |
| Essex and Colchester Hospital ... ... ... ... | 90 | 61½ | 300 0 0 | 225 0 0 | 6 4 | 71 5 0 |
| Cheltenham General Hospital ... ... ... ... | 82 | 58 | — | 127 10 0 | 2 3 | 14 6 11 |
| Staffordshire General Infirmary ... ... ... ... | 75 | 46 | — | 41 15 0 | 6 7½ | 13 15 10 |
| Coventry and Warwickshire Hospital ... ... ... | 80 | 58 | 100 0 0 | 83 0 0 | 6 7½ | 27 2 9 |
| Suffolk General Hospital ... ... ... ... ... | 70 | 44 | 200 0 0 | 166 15 0 | 7 2½ | 60 1 8 |
| Stockport Infirmary ... ... ... ... ... | 60 | 48 | — | 279 0 0 | 4 8 | 65 2 0 |
| Torbay Hospital ... ... ... ... ... ... | 57 | 32 | 10 0 0 | 7 0 0 | 5 8¾ | 2 0 1 |
| Bucks General Infirmary ... ... ... ... ... | 50 | 31 | 120 0 0 | 108 0 0 | 4 9 | 25 13 0 |
| Walsall and District Hospital ... ... ... ... | — | — | 53 0 0 | — | 6 7 | 17 8 11 |
| Stroud General Hospital ... ... ... ... ... | 35 | 23 | — | 38 10 0 | 5 5½ | 10 10 5 |
| North Lonsdale Hospital ... ... ... ... ... | 32 | 15 | 200 0 0 | 170 0 0 | 4 5 | 37 10 10 |
| Carnarvon and Anglesea Infirmary ... ... ... | 28 | 23 | Not | Rated. | — | — |
| Cleveland Cottage Hospital ... ... ... ... | 22 | — | Not | Rated. | — | — |
| Enfield Cottage Hospital ... ... ... ... ... | 10 | 8 | 4 0 0 | 4 0 0 | 6 8 | 1 6 8 |

* Since reduced to £50.

brooke's Hospital, Cambridge, with 106 occupied beds, the Bradford Infirmary with 173 occupied beds, and the Bath Royal Mineral Water Hospital and the Gloucester General Infirmary, with 150 and 92 occupied beds respectively, should be rated at 4s. to 4s. 6d. in the pound. Again, on what principle is a rate of 4s. 6d. in the pound levied on the Birmingham Hospitals and a rate of 7s. 3d. in the pound levied on the Wolverhampton Hospital? Why, again, should the Norfolk and Norwich Hospital have to pay 8s. 8d. in the pound, or the Hull Royal Infirmary 7s. 1d. in the pound, or the Suffolk General Hospital 7s. 2d. in the pound? The differences here shown are sufficient to warrant a close investigation, and we are of opinion that the hospital authorities would be well advised to put their heads together and make a representation to the rating authorities, with a view of securing something like uniformity and a material reduction in the rates at present paid.

No organisation tends to lessen the poor-rate more than the voluntary hospitals. This fact makes it plain that if the hospital authorities will bestir themselves, and bring the facts to the notice of the local authorities, a substantial reduction on the present rates may everywhere be hoped for. We are aware that Parliament is opposed to the abolition of the payment of rates by any class of institution, not excepting the hospitals. It is one thing, however, to maintain a general principle of rating, and quite another to levy a heavy rate upon the voluntary hospitals without any proper regard to the enormous services they render to the ratepayers by providing medical treatment for the poor of all classes. It is within our experience that, when the facts have been properly put before the rating authority in regard to a particular hospital, the vestry or the overseers of the poor, or whoever the rating authority may be, have invariably taken the common-sense view and reduced the assessment, often to a merely nominal amount. This experience leads us to declare that the present rates paid by the hospitals throughout the country may be materially reduced if the various committees and their officers will bestir themselves in the matter, and, having ascertained all the facts, will take steps to bring them to the notice of the rating authority.

